# Two new species of the tribe Synanthedonini (Lepidoptera, Sesiidae), with new hostplant associations from Taiwan

**DOI:** 10.3897/zookeys.861.34387

**Published:** 2019-07-08

**Authors:** Jia-Yuan Liang, Yu-Feng Hsu

**Affiliations:** 1 Department of Life Science, National Taiwan Normal University, Taipei, 116 Taiwan National Taiwan Normal University Taipei Taiwan

**Keywords:** host plants, *
Paranthrenella
*, *
Synanthedon
*

## Abstract

Two new species of the tribe Synanthedonini are described from Taiwan: *Synanthedonauritinctaoidis***sp. nov.** and *Paranthrenellahelvola***sp. nov.** Diagnostic characters for the two species are presented using body color, wing pattern, and genitalia. New records of the relationships between host plants and the immatures are also provided. *S.auritinctaoidis* feeds in the trunk of *Cinnamomumcamphora* (Lauraceae) and *P.helvola* in callus tissue of *Heliciaformosana* (Proteaceae) or *Prunuscampanulata* (Rosaceae).

## Introduction

Clearwing moths (Sesiidae) are a family of small to medium-sized moths that are well-known for their striking mimicry of various Hymenoptera. The Synanthedonini forms the largest tribe in the family Sesiidae Boisduval, 1828 (Špatenka et al. 1999), with 737 species in 38 genera described worldwide (Pühringer and Kallies 2017). The most diverse fauna of clearwing moths, both in species numbers and higher-level diversity, can be found in the tropics and subtropics of the Oriental and Afrotropical regions (Agassiz and Kallies 2018). However, the Synanthedonini fauna of these regions is only incompletely known and many more taxa await discovery or formal description.

Collecting of clearwing moths during the last decades largely relied on artificial pheromone lures (Gorbunov and Arita 1999, 2000; Arita and Gorbunov 2001, 2002; Kallies et al. 2014). However, these lures were produced on the basis of pheromone components identified for a relatively small number of Palaearctic and Nearctic species, and many taxa that occur in the tropical regions do not respond to these lures. Thus, discovery of the host plants and the rearing of larvae are still critical when assessing Sesiidae distribution and diversity. Most clearwing moths are host-specific, as they utilize only one or few related plants as larval hosts (Špatenka et al. 1999; Ahmad et al. 2001; Robinson et al. 2001, 2002). Liang and Hsu (2015) undertook a survey of the family by exploring potential larval host plants, resulting in the discovery of six new species in the tribe Synanthedonini of Taiwan. We continued to employ this investigation strategy, and subsequently two previously unknown Synanthedonini species were discovered in Taiwan, one in the genus *Synanthedon* Hübner, 1819 and the other in *Paranthrenella* Strand, 1916, based on diagnostic characters in body size, wing pattern, and genitalia.

The present article provides the taxonomic treatments and documents host associations, and immature biology for these two species.

## Materials and methods

Adults were collected from flowers or vegetation in the field. Immatures were collected from host plants and reared using an artificial diet. The ingredients of the artificial diet were modified from Liang and Hsu (2015) by adding wood powder of their specific host plant. Forewing length is defined as the distance between the base of the forewing and forewing apex. Genitalia slides were prepared following procedures of Common (1990). Terminology of genitalia follows Klots (1970) and Kristensen (2003), that of wing pattern and venation Špatenka et al. (1999). The names of hostplants follow Boufford et al. (2003). Holotypes will be deposited in the Natural History Museum, London. Additional type series or vouchers are deposited in the following collections abbreviated in the text as follows:

**NHMUK** Department of Entomology, The Natural History Museum, London;

**NTNU** Department of Life Science, National Taiwan Normal University, Taipei, Taiwan.

## Taxonomic accounts

### Synanthedon auritinctaoidis sp. nov.

Taxon classificationAnimaliaLepidopteraSesiidae

http://zoobank.org/FA037243-D2EE-4094-98AB-03EC7A78EA9A

**Figures 1–4.**
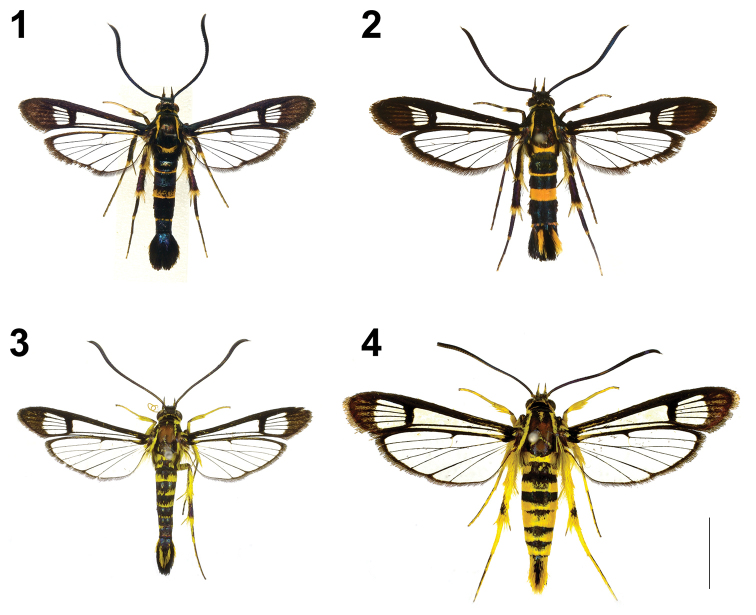
Synanthedonini adults **1, 2***Synanthedonauritinctaoidis* sp. nov. **1** ♂, holotype, Taiwan: Hualien (NHMUK). **2** ♀, paratype, Taiwan: Hualien (NTNU). **3, 4***Paranthrenellahelvola* sp. nov. **3** ♂, holotype, Taiwan: Nantou (NHMUK) **4** ♀, paratype, Taiwan: Nantou (NTNU). Scale bar: 10 mm.

**Figures 5, 6.**
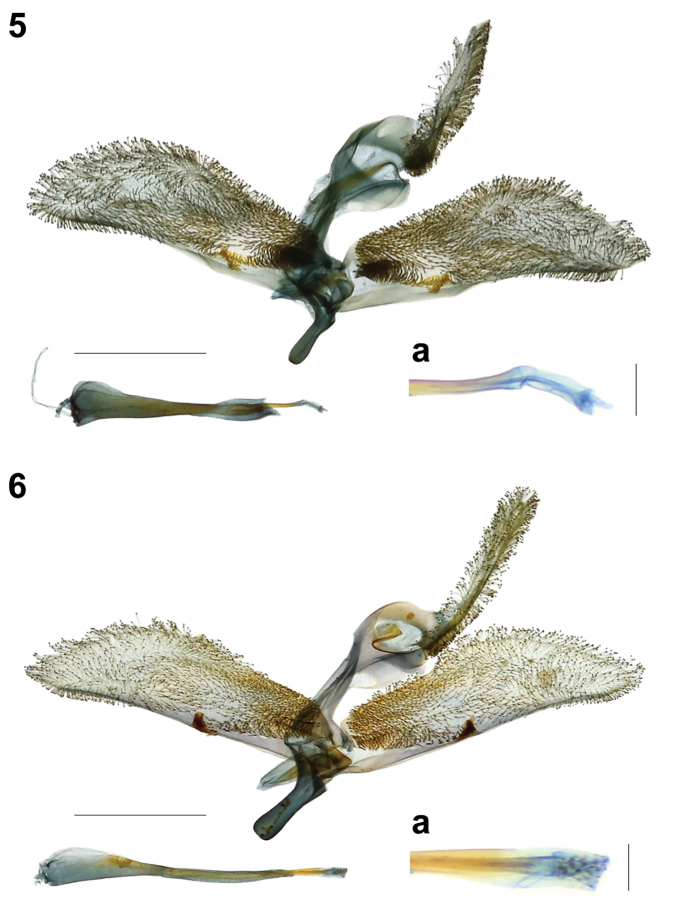
Male genitalia of Synanthedonini. **5***Synanthedonauritinctaoidis* sp. nov. paratype (NTNU) **5a** distal part of phallus. **6***Paranthrenellahelvola* sp. nov. holotype (NHMUK) **6a** distal part of phallus. Scale bars: 1 mm; 0.1 mm (**5a, 6a**).

**Figures 7, 8.**
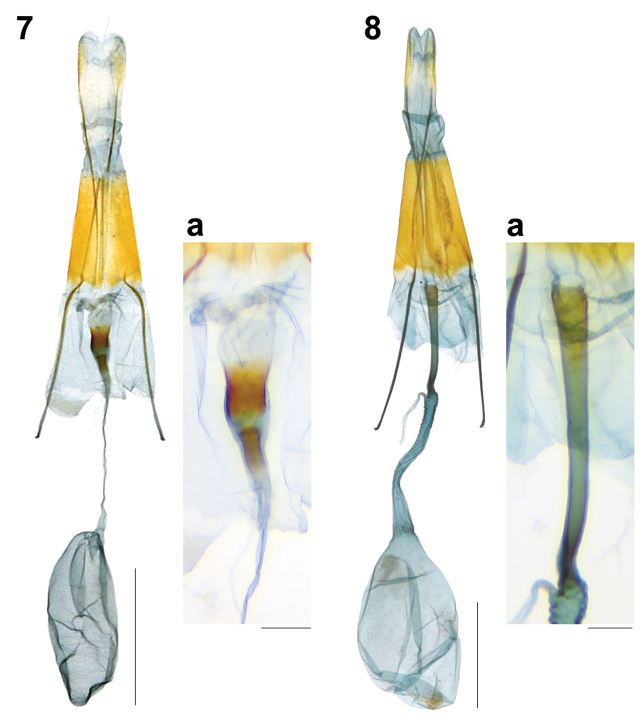
Female genitalia of Synanthedonini. **7***Synanthedonauritinctaoidis* Liang & Hsu, sp. nov. paratype (NTNU) **7a** antrum. **8***Paranthrenellahelvola* Liang & Hsu, sp. nov. paratype (NTNU) **8a** antrum. Scale bars: 1 mm; 0.1 mm (**7a, 8a**).

**Figures 9–12.**
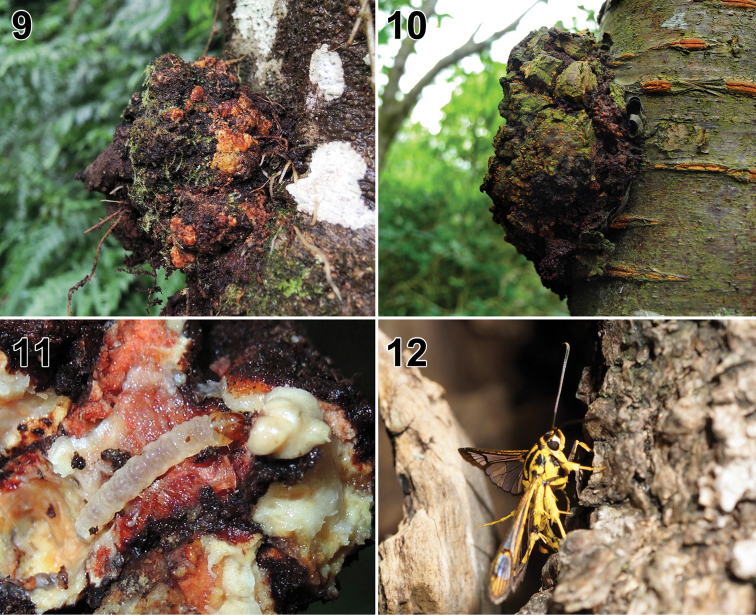
Biology. **9** Galls induced by infection of a *Synanthedonauritinctaoidis*. Caterpillar on burls of *Heliciaformosana* (Hualien Prefecture, Ruisui Township, Fuyuan National Forest Recreation Area) **10** galls induced by infection of a *S.auritinctaoidis* caterpillar on burls of *Prunuscampanulata* (New Taipei City, Shenkeng District, Houshanyue) **11** caterpillar of *S.auritinctaoidis* sp. nov. in burls of *H.formosana* (Hualien Prefecture, Ruisui Township, Fuyuan National Forest Recreation Area) **12** female adult of *Paranthrenellahelvola* sp. nov. ovipositing on bark of *Cinnamomumcamphora* (Taoyuan City, Fuxing District, Lian).

Figs 1 , 2 , 5 , 7 , 9–11

#### Type material.

Holotype: ♂, HUALIEN: Ruisui, Fuyuan National Forest Recreation Area, 410 m, 18 Feb 2018, reared from *Heliciaformosana*, emg. 8 Mar 2014, J.Y. Liang Coll. (NHMUK). Paratypes: 5♀, same locality and date as holotype, emg. 12−24 Mar 2014, J.Y. Liang Coll. (1♀ Gen. Prep. JYL-303) (NTNU); 1♀, same locality, 6 Feb 2016, reared from *H.formosana*, emg. 11 Mar 2016, HSUM 16B82M, J.Y. Liang Coll. (NTNU); 1♂, 4♀, NEW TAIPEI CITY: Shenkeng, Houshanyue, 470 m, 16 Nov 2014, reared from *Prunuscampanulata*, emg. 27 Dec 2014−4 Jan 2015, HSUM 14L07M, J.Y. Liang Coll. (1♂ Gen. Prep. JYL-302) (2♀ Gen. Prep. JYL-271 and JYL-306) (NTNU).

#### Description.

Male (Fig. 1): Antenna length 6.9−7.8 mm (n = 2); forewing length 8.4 −9.2 mm (n = 2); body length 10.7−12.1 mm (n = 2). Head: antenna black with blue-violet sheen; frons white; labial palpus black, yellow ventrally; vertex black with purplish sheen; pericephalic scales yellow with a few black scales dorsally. Thorax: patagia black with violet sheen; tegula black, bronzed-blue sheen with a yellow dorsal line; mesothorax black with blue sheen; metathorax yellow; thorax laterally yellow with a few black scales. Legs: fore coxa externally black, internally yellow; fore femur black, with violet sheen; fore tibia dark brown to black, with admixture of yellow scales distally; fore tarsus dorsally dark brown to black, ventrally entirely yellow; mid coxa and femur black, with violet sheen; mid tibia dark brown to black, base-ventrally with a large yellow spot, base of spurs yellow; spurs yellow with black distally; mid tarsus dorsally dark brown to black, with admixture of yellow scales distally, ventrally yellow; hind leg similar. Abdomen: black with blue sheen; tergites 2 and 6 with a narrow yellow stripe distally; tergite 4 with a broad yellow stripe; abdominal tuft black with bronzed-blue sheen, lateral margins with some yellow-orange scales. Forewing: basally black; costal margin dark brown to black; discal spot and veins within exterior transparent area dark brown to black; apical area dark brown with admixture of brown scales; discal spot broad; exterior transparent area large divided into four cells, level to M2 about 1.5×as broad as discal spot and 0.6× as broad as apical area; posterior transparent area reaching discal spot; cilia dark brown. Hindwing: transparent; veins, discal spot and outer margin dark brown to black with bronzed sheen; discal spot small, cuneiform, reaching to vein M2; cilia dark brown, pale yellow anally.

Female (Fig. 2): Antenna length 5.1−6.2mm (n = 9); forewing length 7.9−8.8 mm (n = 9); body length 8.5−10.8 mm (n = 9). Tergite 4 throughout yellow; anal tuft yellow laterally. Other characters identical to those of male.

Male genitalia (Gen. Prep. JYL-302, NTNU, Fig. 5): Tegumen-uncus complex broad; socii well-developed with scopula androconialis, long, about as short as tegumen-uncus complex; uncus with a small narrow wing ventrally; crista gnathi medialis broad, with distal margin divided in two narrow wings; crista gnathi lateralis consisting of a rather broad, subcordiform, distal part and a narrow, crescent-shaped, proximal part; valva elongated, trapeziform, slightly turned down ventro-caudally; crista sacculi well-developed, large, divided into two pocket-shaped parts; dorsal part larger and armed at distal margin with strong, short, slightly bifurcate distally setae; ventral part of crista sacculi narrow, without setae; saccus rounded basally; phallus thin, about 0.8× as short as valva; vesica without cornuti.

Female genitalia (Gen. Prep. JYL-306, NTNU, Fig. 7): 8^th^ tergite relatively large and broad with a few setae at distal margin; posterior apophysis long, about 2× as long as anterior apophysis; ostium bursae opening near anterior margin of 8^th^ sternite; antrum broad ring, well-sclerotized; ductus bursae narrow, long, membranous; corpus bursae membranous, ovoid, without signum.

#### Diagnosis.

*Synanthedonauritinctaoidis* sp. nov. is similar to *S.auritincta* (Wileman & South, 1918) in markings of body and wing, but may be distinguished by the following genitalia characters: saccus base rounded in *S.auritinctaoidis*, but emarginate in *S.auritincta*; phallus without tooth in *S.auritinctaoidis*, but *S.auritincta* with a small, strong tooth ventro distally; ostium bursae opening near anterior margin of 8^th^ sternite in *S.auritinctaoidis*, but middle of 8^th^ sternite in *S.auritincta*; antrum broad ring in *S.auritinctaoidis*, but funnel-shaped in *S.auritincta*.

#### Etymology.

This species is named *auritinctaoidis*, an adjective formed by adding the suffix –oides to *auritincta*, because of its superficial resemblance with *S.auritincta* (Wileman & South, 1918).

#### Biology.

The larva bores into burls of 5−20 cm in diameter on the trunk or branch of *Heliciaformosana* Hemsl. (Proteaceae) (Figs 9, 11) or *Prunuscampanulata* (Maxim.) Koidz. (Rosaceae) (Fig. 10), and feeds on callus tissue around the hole, which is covered with silk, debris, and frass.

#### Distribution.

Known only from Taiwan.

### Paranthrenella helvola sp. nov.

Taxon classificationAnimaliaLepidopteraSesiidae

http://zoobank.org/996688CE-0504-45CC-B319-3F08ED4114A1

Figs 3 , 4 , 6 , 8 , 12

#### Type material.

Holotype: ♂, NANTOU: Renai, Lushan, 1150 m, 23 Jun 2016, on flower of *Ampelopsisbrevipedunculata*, J.Y. Liang Coll. (Gen. Prep. JYL-301, NHMUK). Paratypes: 1♀, NANTOU: Renai, Aowanda National Forest Recreation Area, 1240 m, 15 Jul 2016, J.Y. Liang Coll. (NYNU); 1♀, TAOYUAN: Fuxing, Lian, 790 m, 22 May 2018, on trunk of *Cinnamomumcamphora*, Y.F. Hsu Coll. (Gen. Prep. JYL-300, NTNU).

#### Description.

Male (Fig. 3): Antenna length 7.2 mm; forewing length 10.3 mm; body length 12.5 mm. Head: antenna black with blue sheen, dorsally and light brown with a few yellow scales ventrally; frons white; labial palpus yellow with black apically; vertex black; pericephalic scales yellow with a few yellow-orange scales. Thorax: patagia dark brown to black, with a small yellow spot laterally; tegula black with a yellow dorsal line, narrowly bordered with yellow scales; thorax laterally yellow with small black scales; mesothorax black with a few yellow scales anteriorly; metathorax yellow, with a few black scales; posteriorly metepimcron and metameron with brown and yellow hair-like scales. Legs: fore coxa yellow with golden sheen, with a broad dark brown to black stripe; fore femur externally dark brown, internally yellow; fore tibia yellow with golden sheen, with admixture of dark brown scales; fore tarsus yellow; mid coxa dark brown to black, with a few yellow scales; mid femur externally dark brown to black, internally yellow; mid tibia yellow, spurs yellow; mid tarsus exterior-dorsally dark brown to black, interior-ventrally entirely yellow; hind leg similar; hind tibia yellow, with a dark brown to black spot distally. Abdomen: dorsally dark brown to black with blue sheen; tergites each with a broad, broadened laterally, yellow stripe distally; abdominal tuft black, dorsal with yellow V shape spot, lateral margins with yellow-orange scales. Forewing: basally black; costal margin dark brown to black, with a narrow yellow stripe between vein Sc and R-stem; discal spot and veins within exterior transparent area dark brown to black; apical area brown with admixture of orange scales; discal spot broad; exterior transparent area large divided into four cells, level to M2 about 3.2× as broad as discal spot and 0.8× as broad as apical area; posterior transparent area reaching distal margin of discal spot; cilia brown. Hindwing: transparent; veins, discal spot black, small, cuneiform, reaching to vein M2; cilia dark brown, yellow anally.

Female (Fig. 4): Antenna length 8.3−8.7 mm (n = 2); forewing length 12.1−12.5 mm (n = 2); body length 13.8−14.1 mm (n = 2). Body and legs with more numerous yellow scales; anal tuft yellow-orange laterally. Color patterns otherwise as in male.

Male genitalia (Gen. Prep. JYL-301, NTNU, Fig. 6): Tegumen-uncus complex narrow; scopula androconialis well-developed, long, about as long as tegumen-uncus complex; crista gnathi medialis relatively long with sinusoid margin; crista gnathi lateralis shorter and narrower than crista gnathi medialis; valva trapeziform-oval, covered with apically bifurcate setae; ventral crista small, covered with triangular flat-topped setae; phallus thin, slightly shorter than valva; vesica with numerous small cornuti.

Female genitalia (Gen. Prep. JYL-300, NTNU, Fig. 8): Apophysis posterioris about as long as apophysis anterioris; ostium bursae opening somewhat anteriorly of 8^th^ tergite; antrum narrow, long, about twice shorter than anterior apophysis, well-sclerotized; ductus seminalis just from anterior margin of antrum; ductus bursae membranous, narrow, slightly longer than antrum, gradually broadened towards corpus bursae; corpus bursae globose to ovoid, without signum.

#### Diagnosis.

*Paranthrenellahelvola* sp. nov. is similar to *P.similis* Gorbunov & Arita, 2000 which was described from Vietnam. *P.helvola* may be distinguished from *P.similis* by forewing without a transparent area of a cell between veins R4 and R5, with a narrow yellow stripe between vein Sc and R-stem; abdominal tuft with yellow V shape spot of male. *P.helvola* possesses wing pattern similar to *P.albipuncta* Gorbunov & Arita, 2000 from Vietnam. *P.helvola* can be distinguished by the coloration of the antenna black, with a large yellow spot in *P.albipuncta*.

#### Etymology.

The name of this new species is the feminine form of the Latin adjective *helvolus*, meaning yellowish, because of the overall yellow coloration of the body trunk and legs of the moth.

#### Biology.

Females lay eggs in the crack of bark (Fig. 12). The larva bores into the trunks of *Cinnamomumcamphora* (L.) (Lauraceae) and feeds on callus tissue around the hole, which is covered with silk, debris, and frass. The adults have been observed taking nectar from flowers of *Ampelopsisbrevipedunculata* (Maxim.) Traut. (Vitaceae) during day time.

#### Distribution.

Known only from Taiwan.

#### Remarks.

The male genitalia of *P.helvola* sp. nov. are similar to those of *P.albipuncta* Gorbunov & Arita, 2000, but the color of the antenna of these two species differs considerably.

## Discussion

Although many plant families are known to be utilized as larval hostplants by sesiid moths, Lauraceae are rarely used, with merely two species recorded: *Paranthrenellaweiyui* Liang & Hsu, 2015 and *Ichneumenopteragryphus* Liang & Hsu, 2015 (Liang and Hsu 2015). *Paranthrenellahelvola*, sp. nov. represents the third known species associated with Lauraceae.

Although most clearwing moths are host-specific, utilizing only one or few related plants as larval hosts, their host range seems to increase with some particular modes of feeding. A wider host range seems also to occur in other groups of insects with particular feeding habits (Nyman et al. 2010). For instance, *Synanthedonscitula* (Harris, 1839), a species feeding exclusively on callus, is known to use 17 genera in nine plant families as larval hosts (Robinson et al. 2002). Larvae of *Synanthedonauritinctaoidis* sp. nov. were found feeding on callus tissue of plants in both Proteaceae and Rosaceae, suggesting more plant families may serve as larval diet for this species.

## Supplementary Material

XML Treatment for Synanthedon auritinctaoidis

XML Treatment for Paranthrenella helvola

## References

[B1] Agassiz D Kallies A (2018) A new genus and species of myrmecophile clearwing moth (Lepidoptera: Sesiidae) from East Africa. Zootaxa 4392(3): 588–594. 10.11646/zootaxa.4392.3.8 29690399

[B2] Ahmad M Mishra R Ahmad J (2001) Insect pest spectrum of Poplar in India. Indian Forester 127(12): 1353−1366.

[B3] Arita Y Gorbunov O (2001) Sesiidae of Taiwan. I. The Tribes Tinthiini, Similipepsini, Paraglosseciini, Pennisetiini, Paranthrenini and Cissuvorini. Japanese Journal of Systematic Entomology 7(2): 131−188.

[B4] Arita Y Gorbunov O (2002) Sesiidae of Taiwan. II. The tribes Osminiini, Melittiini and Sesiini. Japanese Journal of Systematic Entomology 8(2): 199−241.

[B5] Boufford DE Ohashi H Huang TC Hsieh CF Tsai JL Yang KC Peng CI Kuoh CS and Hsiao A (2003) A checklist of the vascular plants of Taiwan. In: Second Edition (Eds) Flora of Taiwan, second edition.Volume six, Department of Botany, National Taiwan University, 15–139.

[B6] Common IFB (1990) Moths of Australia. Melbourne University Press, Carlton, Victoria, 535 pp 10.1071/9780643101227

[B7] Gorbunov O Arita Y (1999) New taxa of the clearwing moths (Lepidoptera, Sesiidae) from Nepal. Tinea 16(2): 106–143.

[B8] Gorbunov O Arita Y (2000) Study on the Synanthedonini (Lepidoptera, Sesiidae) of Vietnam. Japanese Journal of Systematic Entomology 6(1): 85–113.

[B9] Kallies A Arita Y Owada M Wu G-Y Wang M (2014) The Paranthrenini of mainland China (Lepidoptera, Sesiidae). Zootaxa 3811(2): 185–206. 10.11646/zootaxa.3811.2.2 24943158

[B10] Klots AB (1970) Lepidoptera. In: Tuxen SL (Ed.) Taxonomist’s glossary of genitalia in insects. Munksgaard, Copenhagen, 115−130.

[B11] Kristensen NP (2003) Skeleton and muscles: adults. In: Kristensen NP (Ed.) Lepidoptera, Moths and Butterflies, 2 Morphology, physiology and development. Handbook of Zoology 4 (36), De Gruyter, Berlin, New York, 39−131. 10.1515/9783110893724.39

[B12] Liang JY Hsu YF (2015) A review of clearwing moths in the tribe Synanthedonini, with descriptions of six new species from Taiwan (Lepidoptera: Sesiidae). Zootaxa 4044(4): 535–555. 10.11646/zootaxa.4044.4.4 26624724

[B13] Nyman T Vikberg V Smith D Boevé J-L (2010) How common is ecological speciation in plant-feeding insects? A ‘Higher’ Nematinae perspective. BMC Evolutionary Biology 10: 266. 10.1186/1471-2148-10-266 PMC2936908 20807452

[B14] Pühringer F Kallies A (2017) Provisional checklist of the Sesiidae of the world (LepidopteraDitrysia). http://www.sesiidae.net/Checklst.htm [Last modified January 28, 2017; accessed March 8, 2019]

[B15] Robinson GS Ackery PR Kitching IJ Beccaloni GW Hernández LM (2001) Hostplants of the moth and butterfly caterpillars of the Oriental Region. Southdene Sdn Bhd, Kuala Lumpur, 744 pp.

[B16] Robinson GS Ackery PR Kitching IJ Beccaloni GW Hernández LM (2002) Hostplants of the moth and butterfly caterpillars of America north of Mexico. American Entomological Institute 69: 1–824.

[B17] Špatenka K Gorbunov O Laštůvka Z Toševski I Arita Y (1999) Sesiidae, Clearwing Moths. In: Naumann CM (Ed.) Handbook of Palaearctic Macrolepidoptera. Gem Publishing Company, Wallingford, England, 569 pp.

